# Severe Anaphylaxis to Volplex, a Colloid Solution during Cesarean Section: A Case Report and Review

**DOI:** 10.1155/2009/374791

**Published:** 2009-05-26

**Authors:** K. Karri, R. Raghavan, J. Shahid

**Affiliations:** Obstetrics and Gynaecology, Alexandra Hospital, Redditch B98 7UB, UK

## Abstract

Anaphylaxis is a life-threatening event that can occur anytime during pregnancy. It has been reported following administration of various substances with adverse maternal and neonatal consequences. It should be considered in the differential diagnosis of intrapartum collapse. We encountered a case of severe anaphylactic reaction following a routine cesarean section. It is very important that all members of the perinatal team are aware of early recognition and management of anaphylactic reaction. We think that it is important to highlight this as a further case report of severe anaphylactic reaction to a colloid solution and discuss the pathophysiology and management.

## 1. Introduction

Anaphylaxis is an acute systemic reaction with life-threatening consequences. Anaphylaxis although uncommon can lead to adverse maternal and fetal consequences if it occurs during pregnancy. It can be severe, can occur within minutes after initial exposure to the offending agent, and can occur with no history of known allergy. In United States, it has been estimated that 1 to 17% of the population are at risk of anaphylaxis and of these 0.002% are at risk of fatal reactions [1]. Incidence among inpatients has been reported to be 3–5 per 10 000 [2]. The estimated incidence of intra-operative anaphylaxis is between 1:3500 and 1:20 000 [3, 4]. Anaphylaxis can result in significant long-term morbidity, mainly related to cerebral hypoxia after an ineffective resuscitation. It is very important that the members of the perinatal team are aware of the symptoms and signs of anaphylactic reaction and are familiar with the management of anaphylactic shock. We report a case of anaphylactic reaction to Volplex, a commonly used colloid solution, in the immediate postoperative period following cesarean section and reviewed the literature on pregnancy and anaphylaxis.

## 2. Case History

 A 34-year-old primigravida underwent a cesarean section for failed induction of labour under spinal anesthesia. She booked with a Body mass Index of 31. She was diabetic on insulin and was on methyl dopa in view of essential hypertension. The surgery was uneventful until the very end. Towards the end of the procedure, the systolic component of the blood pressure dropped to 85 mm Hg. She was given 500 mL of Volplex, a gelatin-based colloid. Within few minutes, she developed tingling, itching around face including tongue and lips. Then she complained of difficulty in breathing and blood pressure dropped to 65 mm Hg systolic. The colloid was stopped. She was given adrenaline 100 *μ*g intravenously and following that she was given hydrocortisone 100 mg and chlorphenaramine 10 mg. With these medications, the blood pressure improved and there was marked improvement in her symptoms. She was well oxygenated and did not need intubation. She responded well to resuscitation and was transferred to Intensive care unit. Serum tryptase was sent one hour postincident and was 41.6 which is consistent with anaphylaxis. Repeat value of Tryptase on the next day was 11. She had no previous incidents of anaphylaxis and no history of any known allergies. Skin prick testing to ascertain the causative agent was undertaken and anaphylactic reaction due to a gelatin-based colloid was confirmed.

## 3. Pathophysiology

Anaphylaxis is a rapid systemic hypersensitivity reaction to a substance in a sensitised individual with potentially life-threatening consequences. Anaphylaxis is mediated by IgE antibodies, which can cause histamine and other vasoactive mediators to be released from mast cells and basophills [5]. IgE immunoglobulins are found in plasma and are the only antibodies in man to produce anaphylactic reactions, for example, immediate hypersensitivity. The antibodies are generated on exposure to a defined stimulus. These Mast cells are wandering cells that are found in most tissues but are most abundant in connective tissue. These cells liberate histamine in the tissues as part of the inflammatory reaction. These mediators produce respiratory, circulatory, cutaneous, and gastrointestinal effects. Increased vascular permeability and peripheral vasodialation reduce venous return and cardiac output. A mild reaction is manifested as Flushing, urticaria, redness, and localized oedema. More serious reaction is manifested as shock, bronchospasm, laryngeal oedema, and angioedema. Anaphylactic reaction is usually precipitated by blood products, vaccines, insect bites, latex rubber, skin antiseptics, and certain drugs such as antibiotics, Opioid analgesics, and neuromuscular blocking agents. It is more likely to occur after parenteral administration and atopic individuals are particularly susceptible because of their hereditary predisposition to anaphylactic reactions.

## 4. Clinical Manifestations

Symptoms can vary in onset, appearance, and course. Cardiovascular and cutaneous symptoms are more common and account for about three quarters of the manifestations (see Table 1). Mild reactions are manifested in the form of pruritus, rash, flushing of the skin, urticaria, tachycardia, and hypotension. More serious manifestations include urticaria, flush, hypotension, bronchospasm, angioedema, shock, and cardiac arrest. Many present with only one or two of these features. In most cases, the more rapid the onset of symptoms is, the more severe the reaction is. It is very important to stop the administration of the drug or agent at the earliest after the onset of symptoms.

## 5. Diagnosis of Anaphylaxis

History of atopy is present in fewer than 50% of patients. Clinical recognition of manifestations is very important. Serial serum Tryptase estimations are helpful in diagnosis of anaphylaxis [6]. Tryptase enzyme is released from mast cells and it parallels histamine release. Peak concentrations well above 20 ng/mL indicate true anaphylaxis/anaphylactic reaction. The peak value of tryptase in the serum occurs between 30 minutes to 6 hours after an anaphylactic reaction. The value of skin tests, and especially the prick test, has been shown in extensive studies [7]. This demonstrates the presence of specific IgE antibodies. In our reported case, elevated Tryptase levels and skin test confirmed the diagnosis and proved the allergen.

## 6. Treatment

Abrupt cessation of administration of medication is warranted. Treatment should be aimed to maintain good cardiorespiratory support. Epinephrine is the mainstay of the treatment. Hypotension should be corrected by rapid intravascular volume expanders like crystalloids. If colloid is suspected to be the causative agent, it should be replaced by a crystalloid. In patient with cardiovascular collapse, additional doses of epinephrine may be needed. Severe anaphylaxis during antepartum or intrapartum period can result in severe maternal hypotension and this in turn can lead to severe brain damage of the fetus as a consequence of intrapartum hypoxia. Gei et al. reported use of continous infusion of Epinephrine to correct hypotension and fetal bradycardia following anaphylactic shock secondary to Ampicillin during labour [8]. Chaudhuri et al. have reported an allergic reaction to Penicillin in a Primigravida who needed incremental doses of epinephrine for stabilization of blood pressure and the baby was diagnosed with neurological damage [9]. In pregnant patients, continous electronic fetal monitoring is advocated during the event as fetus is very sensitive to maternal changes in blood pressure. The treatment has been summarised in Table 2.

Studies in animals have demonstrated the generation of nitric oxide during anaphylaxis. Inhibition of nitric oxide synthase improves survival in an animal model of anaphylaxis. Nitric oxide causes vasodilation indirectly by increasing the activation of guanylyl cyclase. Methylene blue is an inhibitor of guanylyl cyclase, which increases systemic vascular resistance and reverses shock in animal studies. Treatment with methylene blue should be considered in patients with anaphylactic hypotension that have not responded to other interventions but there have been no reports of its use in the peripartum period [10].

## 7. Discussion

Anaphylactic reactions to anaesthesia and associated agents of anaphylaxis have been reported with increasing frequency [11]. 2000–2002 Confidential Enquiries into Maternal Deaths in the United Kingdom looked into one of the case reports and highlighted the “need for staff to able to manage anaphylaxis.” Volplex is a sterile solution of chemically modified (succinylated) fluid gelatin produced from bovine collagen. This colloid is given as intravenous infusion for the lost blood volume. All available colloid volume substitutes carry the risk of anaphylactic reactions. None of the colloids in clinical use (plasma protein solution, gelatine, hydroxyethylstarch, and dextran) is free from the risk of anaphylactic reactions. Even though the incidence of anaphylactic reactions is low (0.03%), lethal outcome might be encountered. Between the colloids differences exist as far as manifestation (skin, circulatory and respiratory system) and degree of severity of anaphylactic reactions are concerned. Since the underlying pathomechanisms have not been elucidated yet, true prophylactic measures are unknown. Therefore, it is mandatory to control the patient very carefully at the beginning of infusion; early symptoms of anaphylactic reactions should trigger immediate therapeutic measures. Studies of the adverse reactions to gelatin plasma substitutes have concluded that, far from being inert substances, gelatins can initiate a life-threatening anaphylactic response [12]. Allergic reaction to Hydroxyethylstarch (a colloid) has been reported during caesarean delivery [13]. Other substances reported to cause anaphylaxis in pregnancy are Laminaria insertion, Latex, Ranitidine, Antibiotics, sodium ferric gluconate complex, Snake antivenom, insect stings, local anaesthetics, and induction of general anesthesia [14–20].

## 8. Conclusion 

 There must be a written emergency action plan for the management of anaphylaxis. Accurate reporting and issue of Medic-Alert is an important risk management issue. There is no valid predictor of drug anaphylaxis. It is important to analyze circumstances leading to anaphylaxis and maintain fatal anaphylactic register in a national data base [21]. Anaphylaxis can result in significant morbidity mainly related to cerebral hypoxia after an ineffective resuscitation. These reactions can be life threatening in particular if adequate treatment is not started quickly. Since anaphylaxis can occur with any drug, all the members of the perinatal team should be familiar with the recognition of symptoms and signs of anaphylactic reaction. Colloid solutions are used very frequently for postpartum haemorrhage and sometimes antenatally for severe haemorrhage. Anaphylaxis has been reported with commonly used substances like latex, and very commonly used drugs like Oxytocin, antibiotics, and Ranitidine. Colloid solution is a commonly used plasma expander in clinical practice. This case report further highlights the fact that anaphylactic reactions can be unpredictable and severe reactions manifested in the form of angioedema and cardiovascular collapse need immediate and prompt treatment. As seen in the reported case, severe anaphylactic reaction was well managed by the anaesthetist as it happened during a surgical procedure. The importance of awareness among the clinicians about the clinical signs and treatment measures to be undertaken cannot be underestimated as reactions are being observed with commonly used medications. Severe reactions require early recognition and aggressive resuscitation. All obstetricians and midwives should be trained to be able to identify and manage this emergency. The team should also ensure the availability of these drugs in the labour ward as well as in the antenatal and postnatal wards. Every hospital should have a guideline for anaphylaxis. This should be included in the Induction book as part of protocols. Management of anaphylaxis should be included in the emergency drills on a regular basis so that uncommon life-threatening event is tackled in the most effective way.

## Figures and Tables

**Table 1:** Clinical manifestations of drug hypersensitivity [22].

Cardiovascular symptoms	74.7%
Cutaneous symptoms	71.9%
Cardiovascular collapse	50.8%
Bronchospasm	39.8%
Hypotension	17.3%
Angioedema	12.3%
Cardiac arrest	5.9%

**Table 2:** Treatment of anaphylaxis [21].

(1) Stop administration of the drug(s) likely to have caused the reaction.
(2) First line of treatment includes restoration of blood pressure by making the patient lie flat, raising the foot end and lateral tilt of the patient if occurs antepartum.
(3) Maitain airway: give 100% oxygen.
(4) Adrenaline is given intramuscularly 0.5–1.0 mg {0.5–1 mL adrenaline injection 1 in 1000}. This can be repeated every 10 minutes until improvement occurs. If hemodyanamic instability persists, a continuous drip may be needed. One mg of epinephrine is diluted in 250 mL of saline, starts at 15 mL/hr (1*μ*g /mt), and titrated according to clinical response.
(5) Administer crystalloid or colloid for rapid intravascular volume expansion. If colloid has been given prior to the reaction, change to crystalloid since the causative agent might have been the colloid.

Secondary therapy

(1) Antihistamine, for example, Chlorphenaramine over 1 minute 10–20 mg diluted in a syringe with normal saline or water given slowly intravenously.
(2) Corticosteroids (100–500 mg Hydrocortisone IV) is of use in severely affected patients.
(3) Bronchodialators may be required for persistent bronchospasm.
(4) Prolonged monitoring in the Intensive care unit.
